# Effect of recombinant Newcastle disease virus transfection on lung adenocarcinoma A549 cells *in vivo*

**DOI:** 10.3892/ol.2014.2562

**Published:** 2014-09-25

**Authors:** YULAN YAN, LIJUAN JIA, JIN ZHANG, YANG LIU, XUEFENG BU

**Affiliations:** 1 Department of Respiratory Medicine, Affiliated People’s Hospital of Jiangsu University, Zhenjiang, Jiangsu 212002, P.R. China; 2 Department of Internal Medicine, Clinical Medicine College of Jiangsu University, Zhenjiang, Jiangsu 212001, P.R. China; 3 Department of General Surgery, Affiliated People’s Hospital of Jiangsu University, Zhenjiang, Jiangsu 212002, P.R. China

**Keywords:** rl-RVG, oncolytic viruses, immune response, lung cancer

## Abstract

Newcastle disease virus (NDV) has been reported to selectively duplicate in and then destroy tumor cells, whilst sparing normal cells. However, the effect of NDV on lung cancer has yet to be elucidated. In the present study, recombinant NDV (rl-RVG) was applied to lung adenocarcinoma A549 cell tumor-bearing mice to explore its effect on the proliferation of the cells and the immune response of the mice. Following rl-RVG transfection, RVG and NDV gene expression, decreased tumor growth, subcutaneous tumor necrosis, tumor apoptosis and an increased number of cluster of differentiation (CD)3^−^/CD49^+^ natural killer cells were more evident in the rl-RVG group. The present study demonstrated that rl-RVG transfection effectively restrained lung adenocarcinoma A549 cell growth *in vivo*, which may have been accomplish by inducing tumor cell apoptosis and regulating the cell immune response.

## Introduction

Worldwide, lung cancer is the main cause of cancer mortality. Despite the aggressive treatment methods that have been adopted in the past decades, including radiotherapy, chemotherapy and surgery, the five-year survival rate of lung cancer patients remains low.

As one of the novel therapeutic approaches to cancer, oncolytic viral treatments possess great potential. In recent years, it has been found that a variety of viruses possess tumor-oncolytic effects (1). Newcastle disease virus (NDV) is among these oncolytic viruses and has been regarded as a promising oncolytic agent that has been used in experimental clinical therapy for >40 years (2).

NDV has previously been reported to replicate in and kill tumor cells, and since then, researchers have been trying to apply NDV to tumor oncolytic treatments (3). Several clinical trials have proved that NDV is a safe and effective therapeutic agent. The antitumor effect of NDV has been demonstrated in various types of cancers and has shown a significant inhibitory effect on tumor cell growth (4). Human and animal studies have revealed that, subsequent to restructuring, NDV could also be a promising vaccine carrier (5–7). Recombinant NDV strains can be generated using reverse genetics, which results in improved oncolytic and immunoregulatory properties. Rabies virus glycoprotein (RVG), has been revealed to induce the production of neutralizing antibodies and thereby afford complete protection against the challenge of RV (5,8).

The recombinant NDV (rl-RVG) virus applied in the present study, provided by Harbin Veterinary Research Institute (Harbin, Heilongjiang, China), was created with the avirulent NDV LaSota strain and rabies virus glycoprotein (RVG) gene. Human lung adenocarcinoma A549 cell tumor-bearing mice were infected with rL-RVG to study the impact on the proliferation of A549 cell xenograft tumors and the mechanism behind its effect.

## Materials and methods

### Materials

rl-RVG and NDV were provided by Harbin Veterinary Research Institute (Harbin, China). The lung adenocarcinoma A549 cell line, which was purchased from the Cell Culture Center of the Basic Institute of Medical Sciences, Peking Union Medical College (Beijing, China), was reserved for the present experiment. Four-week-old female BALB/c nude mice, were provided by the Comparative Medicine Center of Yangzhou University (Yangzhou, China). Cell culture reagents were acquired from Gibco (Life Technologies, Carlsbad, CA, USA). All polymerase chain reaction (PCR) primers were synthesized by Shanghai Jierui Biotech Co., Ltd. (Shanghai, China). TRIzol reagent was acquired from Fermentas (Burlington, ON, Canada). The PCR master mix was acquired from CWbio Co., Ltd. (Beijing, China). The terminal deoxynucleotidyl transferase-mediated dUTP nick end-labeling (TUNEL) assay kit was acquired from Kaiji Biotech (Nanjing, China). Anti-mouse monoclonal RVG antibodies were purchased from EarthOx (1:500; Millbrae, CA, USA) and anti-chicken monoclonal NDV antibodies were provided by MedImmune, LLC (1:500; Gaithersburg, MD, USA). The polyclonal horseradish peroxidase (HRP)-conjugated goat anti-rabbit and goat anti-mouse antibodies were purchased from Beijing CoWin Biotech, Co., Ltd. (Beijing, China). The HRP-AffiniPure rabbit anti-chicken monoclonal antibody was purchased from EarthOx Life Science (1:10,000; Millbrae). Mouse anti-cluster of differentiation (CD)49b monoclonal antibody was acquired from BioLegend (San Diego, CA, USA).

### Tumor-bearing mouse model construction

Dulbecco’s modified Eagle’s medium supplemented with 10% fetal bovine serum was used to culture the lung adenocarcinoma A549 cells. The cells were incubated at 37°C under a humidified atmosphere of 95% air and 5% CO_2_. At 90% confluence, the A549 cells were reaped and resuspended in phosphate-buffered saline (PBS) at 8.0×10^6^ cells/ml. The tumor-bearing mouse model was constructed by subcutaneous injection of 2.0×10^6^ cells along the left oxter of four-week-old female BALB/c mice. A tumor between 5 and 20 mm in diameter was assessed in each mouse after ~10 days. All tumor-bearing mice were divided randomly into three groups, the PBS, NDV and rl-RVG groups.

### Animal experiment

When the tumors reached 5–20 mm in diameter, the tumors of each mouse were injected with 300 μl PBS in the control PBS group, 6.3×10^8^ pfu NDV in the NDV group and 6.3×10^8^ plaque forming units rl-RVG in the rl-RVG group. Viral transfection was performed twice a week for three weeks. The volume of the tumor was measured at zero, seven, 14 and 21 days. After 21 days, all mice were anesthetized and sacrificed, and the tumor, lung and spleen tissues were split by blunt dissection. Certain tissues were fixed in 4% paraformaldehyde and others were stored at −80°C for subsequent analysis. This study was approved by the Laboratory Animal Management Committee of Jiangsu University (Zhenjiang, China).

### Growth curve of tumor and inhibition rates

When the diameter of the tumor had reached 5–20 mm, Vernier calipers were used to measure the short (a) and long (b) diameters of the tumor every seven days. A growth curve of the tumor volume was drawn according to the formula: Volume = a^2^ × b × 0.52. The tumor inhibition rates were counted based on the last measurement of tumor volume according to the following formula: Tumor inhibition rate = (average tumor volume in PBS group − average tumor volume in treatment group) / mean tumor volume in PBS group × 100.

### Morphological analysis

The tumor, spleen and lung tissues were fixed in 4% paraformaldehyde, embedded in paraffin, sliced into 5-μm thick sections and stained following a hematoxylin and eosin staining procedure. Under an optical microscope (Eclipse TS100; Nikon Corporation, Tokyo, Japan), pathological changes were detected in the tissue sections and images were captured for documentation.

### Reverse transcription (RT)-PCR analysis

PCR was used to determine the expression of the RVG and NDV genes. The total RNA was extracted from the sections of tumor tissue using TRIzol reagent (Thermo Fisher Scientific, Waltham, MA, USA). The cDNA was synthesized using Oligo (dT) primers (Takara Bio, Inc, Otsu, Japan) and reverse transcriptase (Takara Bio, Inc.). The primers for the NDV hemagglutinin-neuraminidase (HN) gene were 5′-CTGGACGGTTTGGTGGGAA-3′ and 5′-TAATGCGACTGCGGGATGTG-3′. The primers for the glycoprotein (G) gene were 5′-AGCCGATGCTCACTACAAG-3′ and 5′-CTGGAGGAGGGATGATTGC-3′. The PCR protocol was as follows: An initial denaturation at 94°C for 5 min, then denaturation at 94°C for 30 sec, annealing at 53°C (RVG) or 55°C (NDV) for 30 sec, and extension at 72°C for 30 sec, for 30 cycles. An incubation step was executed in the final extension at 72°C for 10 min. Electrophoresis of the PCR products was performed in agarose gel, and the results were visualized using ethidium bromide. The bands were analyzed with Quantity One software (Bio-Rad, Hercules, CA, USA).

### Western blotting

Western blotting was used to confirm the expression of the RVG and NDV proteins. Tumor tissue (1 g) was cut into sections and homogenized on ice. Following rapid centrifugation at 12,00 × g, the supernatant was discarded and the pellet was resuspended with 1,000 μl pre-cooled radioimmunoprecipitation assay lysis buffer (3 μl sodium orthovanadate, 3 μl phenylmethyl sulfonyl fluoride and 3 μl protease inhibitor cocktail; KangChen Bio-tech, Inc., Shanghai, China). The mixture was homogenized and lysed for 60 min on ice. Following centrifugation at 12,000 × g for 15 min at 4°C, the supernatant was transferred into a 1.5 ml tube and mixed with an equal volume of loading buffer (2X) and β-mercaptoethanol (1:20). The extracted protein was separated on a 10% SDS-PAGE gel, and the NDV and RVG protein expression levels were determined through use of the indicated primary antibodies (1:500) for 1–1.5 h. Following two washes with PBS, HRP-conjugated secondary immunoglobulin (Ig)G antibodies (1:10,000) were used, then samples were washed with PBS twice. GAPDH was used as a negative control and internal reference.

### Immunohistochemistry test

The tumor tissues were fixed with 4% paraformaldehyde, embedded in paraffin and sliced into 5-μm thick sections. Sections were then placed in dimethylbenzene and a gradient of ethanol (100, 95, 90, 80 and 70%), for 5 min each, for dewaxing, followed by blocking with serum. The samples were incubated with primary antibodies for RVG and NDV (1:200) for three hours each at 37°C and then washed with PBS three times. All sections were incubated with the HRP-conjugated secondary IgG antibodies (1:10,000) for 30 min at 37°C. Subsequent to being washed three times with PBS, the samples were stained with 3,3-diaminobenzidine, kept at room temperature without light for 10 min and then stained with hematoxylin. The samples were then dehydrated using an ethanol gradient (70, 80, 90, 95 and 100%), then rinsed in xylene for 10 min twice. The sections were observed and images were captured under an optical microscope.

### Apoptosis assay

A TUNEL assay kit was used for the analysis of apoptosis according to the manufacturer’s instructions. The sections were analyzed and the images captured under an optical microscope. The apoptosis index was calculated as follows: The number of apoptotic cells / (the number of apoptosis cells + the number of non-apoptotic cells) × 100.

### Flow cytometry

The number of CD3^−^/CD49^+^ natural killer (NK) cells was detected using flow cytometry. Splenocyte suspensions were prepared from the spleens of the sacrificed mice. Splenocytes (100 μl aliquots) were labeled with 0.5 μl CD49b-fluorescein isothiocyanate and 2.5 μl hamster CD3e-phycoerythrin, respectively. The cells were analyzed with a FACSCalibur flow cytometer (BD Biosciences, Franklin Lakes, NJ, USA) using the CellQuest (BD Biosciences) and WinMDI 2.9 (Scripps Research Institute, La Jolla, CA, USA) software.

### Statistical analysis

SPSS 19.0 software (IBM Corp., Armonk, NY, USA) was used to analyze all data and the results were reported as the mean ± standard deviation. One-way analysis of variance was used to analyze the statistical significance, followed by post-hoc comparisons to compare the differences between multiple groups. P<0.05 was considered to indicate a statistically significant difference.

## Results

### Growth curve of tumor and inhibition rates

**Figure 1:**
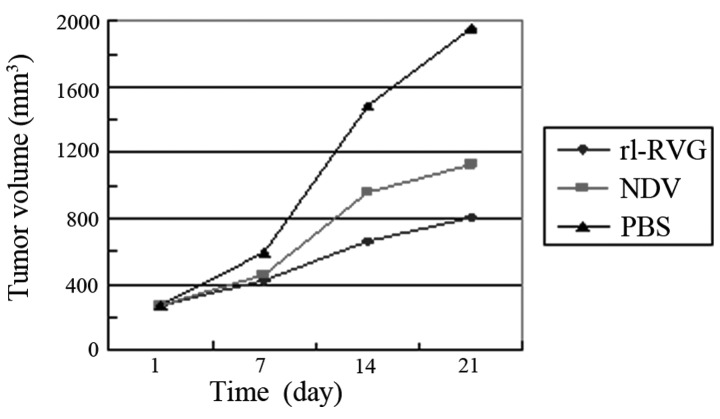
Growth curves of the tumors present in the rl-RVG, NDV and PBS groups. Tumor inhibition rate of rl-RVG = (1960.92 − 804.96) / 1960.92 × 100 = 58.9%. Tumor inhibition rate of NDV = (1960.92–1127.88) / 1960.92× 100 = 42.5%. NDV, Newcastle disease virus; RVG, rabies virus glycoprotein; rl-RVG, recombinant NDV; PBS, phosphate-buffered saline.

The tumor volume in the PBS group continued to increase throughout the 21-day period. In the rl-RVG and NDV groups, the volumes were markedly smaller at day 21 compared with those detected in the PBS group (P<0.01), and the tumor volumes in the rl-RVG group were smaller compared with the NDV group (P<0.05). The tumor inhibition rates were calculated based on the following formula: Volume = a^2^ × b × 0.52. The tumor inhibition rate of rl-RVG was revealed to be 58.9%, which meant that the growth of the tumor in the rl-RVG group was inhibited by 58.9% compared with the PBS group. The tumor inhibition rate of NDV was 42.5%, meaning that the growth of the tumor in the NDV group was inhibited by 42.5% compared with the PBS group (Fig. 1; Table I).

### Morphological and histopathological analysis

The effect of the various treatments on the morphology of the tissue samples from the tumor, lung and spleen were compared. The results revealed that tumor cells from the rl-RVG group underwent the most destructive necrosis, while necrosis was not observed in the PBS group.

In the lung tissue from the rl-RVG and NDV groups, there was a severe inflammatory reaction, while there was no inflammation in the lung tissue from the PBS group. The inflammatory reaction was more severe in the rl-RVG group compared with the NDV group.

**Figure 2:**
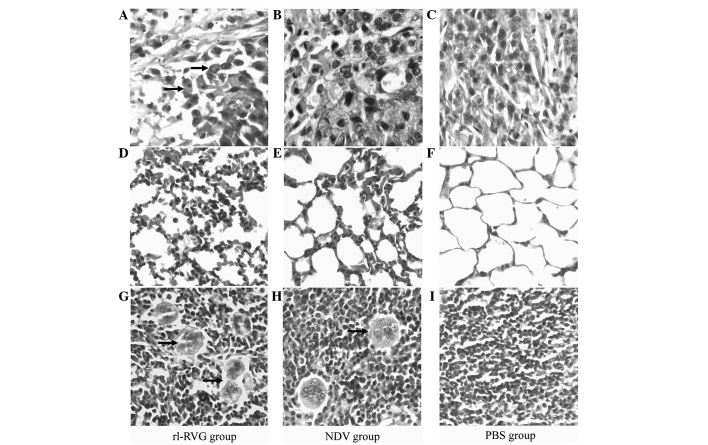
Hematoxylin and eosin-stained tissue sections from the (A–C) tumor, (D–F) lung and (G–I) spleen demonstrating histopathological changes following transfection. (A–C) A larger quantity of subcutaneous tumor necrosis (blue arrows) is present in the tumor tissue from the rl-RVG group. (D–F) A more severe inflammatory reaction occurred in the lung of the mice in the rl-RVG group. (G–I) A larger quantity of multinucleated giant cells (red arrows) are present in the spleen tissue from the rl-RVG group (magnification, ×200). NDV, Newcastle disease virus; RVG, rabies virus glycoprotein; rl-RVG, recombinant NDV; PBS, phosphate-buffered saline.

The spleen tissues exhibited a significant increase in size in the rl-RVG and NDV groups of mice compared with the PBS group, and the spleen tissues were larger in the rl-RVG group compared with those in the NDV group. In the spleen, rl-RVG transfection induced more aggregation of multinucleated giant cells (Fig. 2).

### Expression of the G and NDV genes

**Figure 3:**
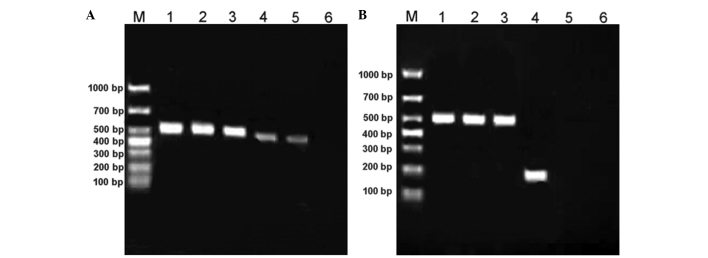
Expression of the (A) NDV and (B) RVG genes in the tumor tissues. (1) rl-RVG group, GAPDH; (2) NDV group, GAPDH; (3) PBS group, GAPDH; (4) rl-RVG group; (5) NDV group; (6) PBS group; (M) Marker. The results display clear (A) NDV gene expression (~462 bp) and (B) RVG gene expression (~176 bp) following transfection with rl-RVG. NDV, Newcastle disease virus; RVG, rabies virus glycoprotein; rl-RVG, recombinant NDV; PBS, phosphate-buffered saline.

RT-PCR was used to detect RVG and NDV gene expression in the tumor tissue. The results revealed clear RVG gene expression (~176 bp) following transfection with rl-RVG. In the PBS and NDV groups, the RVG was not expressed. The NDV gene (~462 bp) was expressed in the rl-RVG and NDV groups, but was not expressed in the PBS group (Fig. 3).

### Expression of the G and NDV proteins

#### Western blotting

**Figure 4:**
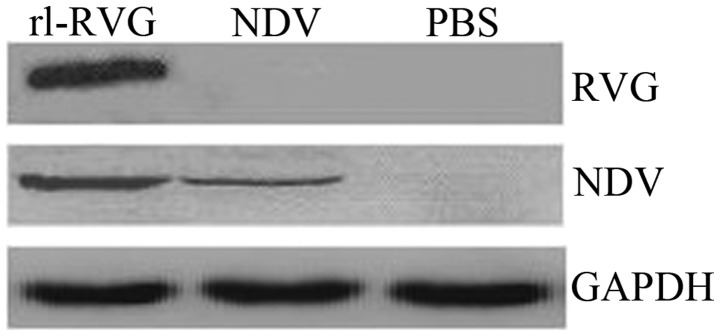
RVG was expressed in the rl-RVG group following transfection with rl-RVG, but in the PBS and NDV groups, RVG was rarely expressed. NDV was expressed in the rl-RVG and NDV groups, but was rarely expressed in the PBS group. NDV, Newcastle disease virus; RVG, rabies virus glycoprotein; rl-RVG, recombinant NDV; PBS, phosphate-buffered saline.

Western blot analysis was used to determine RVG and NDV protein expression. The results revealed that RVG was expressed following transfection with rl-RVG, whereas in the PBS and NDV groups, RVG was not expressed. NDV was expressed in the rl-RVG and NDV groups but was not expressed in the PBS group (Fig. 4).

#### Immunohistochemistry

**Figure 5:**
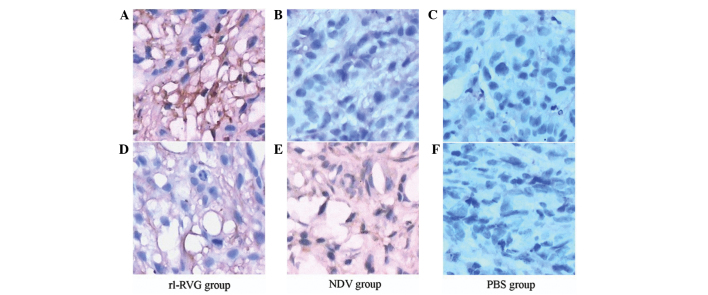
(A–C) The cytoplasm of cells from the rl-RVG group was positive for RVG expression, but the cytoplasm from the PBS and NDV groups was not. (D–F) The cytoplasm of cells from the rl-RVG and NDV groups was positive for NDV expression, and the expression in the rl-RVG group was stronger compared with the NDV group. The PBS group exhibited a negative result for NDV expression (magnification, ×200). NDV, Newcastle disease virus; RVG, rabies virus glycoprotein; rl-RVG, recombinant NDV; PBS, phosphate-buffered saline.

Immunohistochemistry with primary antibodies for NDV and RVG was used to confirm the expression of the NDV and RVG proteins in the tumor tissues. The results revealed that, in the rl-RVG group, RVG was expressed in the tumor cell cytoplasm, but there was no RVG expression in the NDV and PBS groups (Fig. 5A_1_–A_3_). The cytoplasm of the cells from the rl-RVG and NDV groups was positive for RVG expression, and the rl-RVG group exhibited stronger expression compared with the NDV group, while the PBS group was negative for RVG expression (Fig. 5B_1_–B_3_). These results indicated that the NDV proteins were expressed in the cytoplasm of the tumor cells in the rl-RVG and NDV groups and were expressed to a greater extent in the rl-RVG group. Additionally, there were notably more mucous lakes in the RVG group (Fig. 5).

### Apoptosis of tumor cells

**Figure 6:**
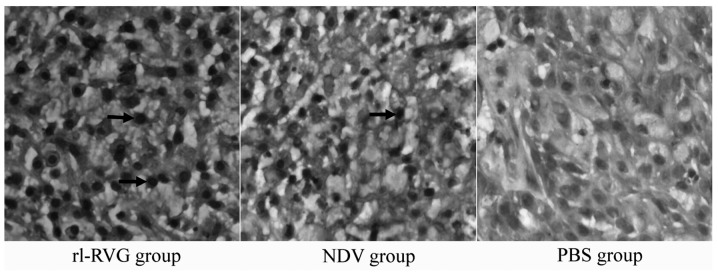
Apoptosis in tumors transfected with rl-RVG, NDV or PBS. The results reveal that the number of apoptotic cells (arrows) and the apoptotic index were markedly higher in the rl-RVG and NDV groups than in the PBS group (P<0.01) and that those of the rl-RVG group were higher compared with the NDV group (P<0.05; magnification, ×200). NDV, Newcastle disease virus; RVG, rabies virus glycoprotein; rl-RVG, recombinant NDV; PBS, phosphate-buffered saline.

A TUNEL assay was performed to detect apoptosis in the tumor cells. The results revealed that the number of apoptotic cells and the apoptotic index were markedly higher in the rl-RVG and NDV groups compared with the PBS group (P<0.01), with the rl-RVG group exhibiting a higher number and index compared with the NDV group (P<0.05) (Fig. 6; Table II).

### Number of NK cells in the spleen

**Figure 7:**
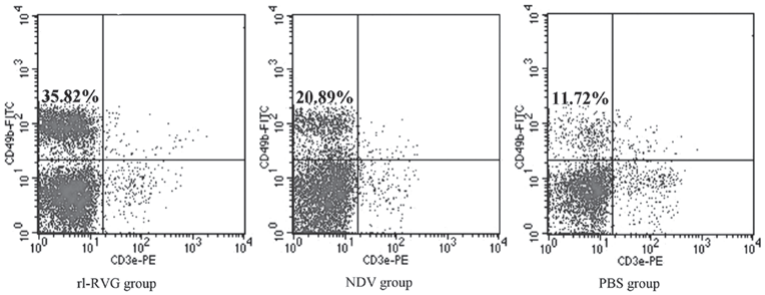
Flow cytometry determined the number of NK cells in the spleen. The results proved that the number of NK cells was markedly higher in the rl-RVG and NDV groups compared with the PBS group (P<0.001), with the rl-RVG group exhibiting a higher number of cells than the NDV group. NK, natural killer; NDV, Newcastle disease virus; RVG, rabies virus glycoprotein; rl-RVG, recombinant NDV; PBS, phosphate-buffered saline; CD, cluster of differentiation; PE, phycoerythrin; FITC, fluorescein isothiocyanate.

Flow cytometry was used to determine the number of NK cells. The results proved that the number of NK cells was markedly higher in the rl-RVG and NDV groups compared with the PBS group (P<0.001), with the rl-RVG group exhibiting the highest levels (Fig. 7; Table III).

## Discussion

Lung cancer is one of the most common malignant tumors and its incidence is increasing. The majority of lung cancer patients have already lost the opportunity to undergo surgery prior to obtaining a clear diagnosis. Also, radiation and chemotherapy can produce evident side-effects and treatment resistance. Oncolytic therapy is one of the most widely researched novel biological treatments. Oncolytic viral therapy is a method that harnesses the natural ability of a virus to infect, duplicate within and lyse a host cell as part of its natural life cycle (1).

A variety of viruses have been revealed to possess oncolytic, antitumoral activity, including herpes simplex virus type 1, vaccinia virus and adenovirus (9).

NDV is a type of fowl cholera virus that mainly infects poultry. NDV is a non-segmented, negative-sense, single-stranded RNA virus of the Paramyxoviridae family, with a natural avian host range. The NDV genome codes for the following six genes, listed in order from the 3′-end: Nucleocapsid protein, phosphoprotein, matrix protein, fusion protein, HN and large protein (5,8).

NDV has been reported to selectively duplicate in and destroy tumor cells, while sparing normal cells, and therefore its application as a oncolytic agent in cancer treatment has been explored (10–14). Immunotherapy using NDV has also been used for the treatment of neuroblastoma (15), melanoma (16,17) and other malignancies. However, to the best of our knowledge, there have been no studies on the anti-lung carcinoma effect of RVG expressing the recombinant avirulent NDV LaSota strain.

RV, a highly neurotropic virus, leads to deadly encephalomyelitis in almost all mammals, including humans. The genome of RV encodes five structural proteins, namely, nucleoprotein, phosphoprotein, matrix protein, G protein and large protein. RVG, a type I transmembrane protein, consists of cytoplasmic, transmembrane and external domains that are exposed as trimers on the surface of the mature virus particle (18). The external domain alone has been revealed to induce the production of neutralizing antibodies and thereby provide complete protection against RV (19). Rabies viruses can spread to contiguous or non-contiguous cells, which are encompassed by the interstitial space. Despite being merged into the surface of NDV virions, RVG does not alter the trypsin-dependent infectivity of NDV in mammalian cell cultures. RVG expression does not affect the initiation of the innate immune response to NDV in mammalian cells. RVG gene expression does not augment the toxicity of the NDV vector in poultry or mice (20). In the present study, rL-NDV was used, which was generated using the RVG gene from a non-pathogenic RV, the ERA strain. Numerous animal studies have revealed that rL-RVG is safe in mice, poultry, dogs and cats (20–23).

It has been demonstrated that the majority of oncolytic NDV strains induce apoptosis through the extrinsic and intrinsic caspase-dependent cell death pathways (24). In the present study, the TUNEL assay demonstrated that the number of apoptotic cells and the apoptotic index were markedly higher in the rl-RVG and NDV groups than in the PBS group, and that the levels in the rl-RVG group were higher than in the NDV group. NDV can duplicate more rapidly in human tumor cells compared with normal cells and then exert oncolytic effects (25). This selective effect is likely due to the restrained production of V proteins and virus-induced cytokines in the host (26). NDV can infect the majority of tumor cells, and the viral duplication in the cells can be tested by detecting the augmentation of viral antigens on the cell surface (27). In the present study, the analysis revealed that the tumor volume was clearly decreased following rl-RVG and NDV transfection.

Despite leading to direct oncolytic effects on tumor cells, NDV can modulate the human immune system. It has been reported that NDV stimulates host immunity to generate cytokines, including interferon (IFN)-β, IFN-α, interleukin (IL)-1 and TNF-α, which in turn, leads to the activation of macrophages, sensitized T cells and NK cells (28). NDV augments antitumor cytotoxic activity through activation of human NK cells. HN is a potent inducer of IFN production by human peripheral blood mononuclear cells and is able to upregulate TNF-related apoptosis inducing ligands (TRAIL) (29). The direct interaction between the HN viral glycoprotein and sialic acid residues on the cell surface could activate NK cells. Thus, those secreted cytokines, including IL-2, IFN-γ and TNF-α, could be stimulated by activated NK cells, further activating and affecting other immune cell functions. It can therefore be speculated that activated NK cells have increased cytolytic antitumor effects (20). The analysis in the present study revealed that tumor necrosis, spleen size, generation of multinucleated giant cells in the spleen and the number of NK cells were markedly increased in the rl-RVG and NDV groups compared with the control group. This phenomenon was more pronounced in the rl-RVG group.

The present study aimed to assess the inhibitory effect of rl-RVG on lung adenocarcinoma A549 cells in tumor-bearing mice and its likely mechanism. The analysis revealed that following rl-RVG or NDV transfection, the tumor volumes were markedly decreased. Immunohistochemistry indicated that in the rl-RVG group, RVG was expressed in the tumor cell cytoplasm, while there was no RVG expression in the NDV or PBS groups; in the rl-RVG and NDV groups, NDV was expressed in the tumor cell cytoplasm, and was expressed to a greater extent in the rl-RVG group compared with the NDV group. There was no NDV expression in the PBS group. RT-PCR and western blotting suggested that the rl-RVG vector was successfully transfected into the adenocarcinoma A549 cells in the tumor-bearing mice, as an increase in the RVG gene and protein expression in the rl-RVG group was observed, and expression of the NDV gene and protein was observed in the rl-RVG and NDV groups. The level of tumor necrosis increased and the spleen became enlarged, with multinucleated giant cell formations. Flow cytometry indicated that the number of NK cells was markedly higher in the rl-RVG and NDV groups than in the control group. This phenomenon was more pronounced in the rl-RVG group. TUNEL assay demonstrated that the apoptotic cell number and the apoptotic index were markedly higher in the rl-RVG and NDV groups compared with the PBS group, with the rl-RVG group exhibiting a higher apoptotic index compared with the NDV group.

In conclusion, the results of the present study indicated that rl-RVG inhibits the growth of lung cancer cells and accelerates apoptosis to a certain extent. rl-RVG can modulate the immune system and strengthen the cell immune response, leading to an anti-tumor effect. The present study is expected to provide an experimental basis for further clinical application of rl-RVG in lung cancer therapy.

## Figures and Tables

**Table I:** Comparison of the tumor volume in nude mice.

Time, days	rl-RVG	NDV	PBS	F-value	P-value
1	274.67±114.20	274.08±127.05	271.78±110.79	0.001	0.999
7	444.18±111.22	453.65±164.94	591.45±244.50	0.846	0.458
14	657.56±176.74	958.26±274.09	1483.21±446.75^a^	7.752	0.009
21	804.96±176.74	1127.88±274.09^c^	1960.92±446.758^b^	26.044	<0.001

a rl-RVG and NDV vs. PBS, P<0.05;

b rl-RVG and NDV vs. PBS, P<0.05;

c rl-RVG vs. NDV, P<0.05.

NDV, Newcastle disease virus; RVG, rabies virus glycoprotein; rl-RVG, recombinant NDV; PBS, phosphate-buffered saline.

**Table II:** AI of tumors in mice transfected with rl-RVG.

Group	AI (n=10)	F-value	P-value
rl-RVG	0.300±0.0408^a^		
NDV	0.225±0.0470^b^	120.249	<0.001
PBS	0.044±0.0212		

a rl-RVG vs. NDV and PBS, P<0.05;

b NDV vs. PBS, P<0.05.

AI, apoptosis index; NDV, Newcastle disease virus; RVG, rabies virus glycoprotein; rl-RVG, recombinant NDV; PBS, phosphate-buffered saline.

**Table III:** Ratio of natural killer cells transfected with rl-RVG.

T cell	rl-RVG	NDV	PBS	F-value	P-value
CD49^+^	43.2±5.26^a^	28.8±2.38^b^	18.8±5.90	31.58	<0.001

a rl-RVG vs. NDV and PBS, P<0.001;

b NDV vs. PBS, P<0.001.

NDV, Newcastle disease virus; RVG, rabies virus glycoprotein; rl-RVG, recombinant NDV; PBS, phosphate-buffered saline.
